# Science without publication paywalls: cOAlition S for the realisation of full and immediate Open Access

**DOI:** 10.1371/journal.pbio.3000031

**Published:** 2018-09-04

**Authors:** Marc Schiltz

**Affiliations:** President, Science Europe, Brussels, Belgium

## Abstract

In this Perspective, a group of national funders, joined by the European Commission and the European Research Council, announce plans to make Open Access publishing mandatory for recipients of their agencies’ research funding.

## Open access is foundational to the scientific enterprise

Universality is a fundamental principle of science (the term ‘science’ as used here includes the humanities): only results that can be discussed, challenged, and, where appropriate, tested and reproduced by others qualify as scientific. Science, as an institution of *organised criticism*, can therefore only function properly if research results are made openly available to the community so that they can be submitted to the test and scrutiny of other researchers. Furthermore, new research builds on established results from previous research. The chain, whereby new scientific discoveries are built on previously established results, can only work optimally if all research results are made openly available to the scientific community.

Publication paywalls are withholding a substantial amount of research results from a large fraction of the scientific community and from society as a whole. This constitutes an absolute anomaly, which hinders the scientific enterprise in its very foundations and hampers its uptake by society. Monetising the access to new and existing research results is profoundly at odds with the ethos of science [1]. There is no longer any justification for this state of affairs to prevail and the subscription-based model of scientific publishing, including its so-called ‘hybrid’ variants, should therefore be terminated. In the 21st century, science publishers should provide a service to help researchers disseminate their results. They may be paid fair value for the services they are providing, but **no science should be locked behind paywalls!**

## A decisive step towards the realisation of full open access needs to be taken now

Researchers and research funders have a collective duty of care for the science system as a whole. The 2003 *Berlin Declaration* [2] was a strong manifestation of the science community (researchers and research funders united) to regain ownership of the rules governing the dissemination of scientific information. Science Europe established principles for the transition to Open Access in 2013 [3] but wider overall progress has been slow. In 2016, the EU Ministers of science and innovation, assembled in the Competitiveness Council, resolved that all European scientific publications should be immediately accessible by 2020.

As major public funders of research in Europe, we have a duty of care for the good functioning of the science system (of which we are part), as well as a fiduciary responsibility for the proper usage of the public funds that we are entrusted with. As university and library negotiation teams in several countries (e.g. Germany, France, Sweden) [4,5] are struggling to reach agreements with large publishing houses, we feel that a decisive move towards the realisation of Open Access and the complete elimination of publication paywalls in science should be taken now. The appointment of the Open Access Envoy by the European Commission has accelerated this process.

Hence, driven by our duty of care for the proper functioning of the science system, we have developed **Plan S** whereby **research funders will mandate that access to research publications that are generated through research grants that they allocate, must be fully and immediately open and cannot be monetised in any way** (Box 1).

**Box 1. Plan S. Accelerating the transition to full and immediate open access to scientific publications**

The key principle is as follows:

"**After 1 January 2020 scientific publications on the results from research funded by public grants provided by national and European research councils and funding bodies, must be published in compliant Open Access Journals or on compliant Open Access Platforms**."

In addition:

- Authors retain copyright of their publication with no restrictions. All publications must be published under an open license, preferably the Creative Commons Attribution Licence CC BY. In all cases, the license applied should fulfil the requirements defined by the Berlin declaration;
- The Funders will ensure jointly the establishment of robust criteria and requirements for the services that compliant high quality Open Access journals and Open Access platforms must provide;
- In case such high quality Open Access journals or platforms do not yet exist, the Funders will in a coordinated way provide incentives to establish these and support them when appropriate; support will also be provided for Open Access infrastructures where necessary;
- Where applicable, Open Access publication fees are covered by Funders or universities, not by individual researchers; it is acknowledged that all scientists should be able to publish their work Open Access even if their institutions have limited means;
- When Open Access publication fees are applied, their funding is standardised and capped (across Europe);
- Funders will ask universities, research organisations, and libraries to align their policies and strategies, notably to ensure transparency;
- The above principles shall apply to all types of scholarly publications, but it is understood that the timeline to achieve Open Access for monographs and books may be longer than 1 January 2020;
- The importance of open archives and repositories for hosting research outputs is acknowledged because of their long-term archiving function and their potential for editorial innovation;
- The ‘hybrid’ model of publishing is not compliant with the above principles;
- The Funders will monitor compliance and will sanction non-compliance.

## Further considerations

We recognise that researchers need to be given a maximum of freedom to choose the proper venue for publishing their results and that in some jurisdictions this freedom may be covered by a legal or constitutional protection. However, our collective duty of care is for the science system as a whole, and researchers must realise that they are doing a gross disservice to the institution of science if they continue to report their outcomes in publications that will be locked behind paywalls.

We also understand that researchers may be driven to do so by a misdirected reward system which puts emphasis on the wrong indicators (*e*.*g*. journal impact factor). We therefore commit to fundamentally revise the incentive and reward system of science, using the San Francisco Declaration on Research Assessment (DORA) [6] as a starting point.

The subscription-based model of scientific publishing emerged at a certain point in the history of science, when research papers needed extensive typesetting, layout design, printing, and when hardcopies of journals needed to be distributed throughout the world. While moving from print to digital, the publishing process still needs services, but the distribution channels have been completely transformed. There is no valid reason to maintain any kind of subscription-based business model for scientific publishing in the digital world, where Open Access dissemination is maximising the impact, visibility, and efficiency of the whole research process. Publishers should provide services that help scientists to review, edit, disseminate, and interlink their work and they may charge fair value for these services in a transparent way. The minimal standards for services expected from publishers are laid down on page 6 of the 2015 ‘Science Europe Principles on Open Access Publisher Services’ [3].

Obviously, our call for *immediate* Open Access is not compatible with any type of embargo period.

We acknowledge that 'transformative' type of agreements, where subscription fees are offset against publication fees, may contribute to accelerate the transition to full Open Access. Therefore, it is acceptable that, during a transition period that should be as short as possible, individual funders may continue to tolerate publications in ‘hybrid’ journals that are covered by such a 'transformative' type of agreement. There should be complete transparency in such agreements and their terms and conditions should be fully and publicly disclosed.

We are aware that there may be attempts to misuse the Open Access model of publishing by publishers that provide poor or non-existent editorial services (*e*.*g*. the so-called ‘predatory’ publishers). We will therefore support initiatives that establish robust quality criteria for Open Access publishing, such as the Directory of Open Access Journals (DOAJ) (https://doaj.org) and the Directory of Open Access Books (DOAB) (https://www.doabooks.org).

We note that for monographs and books the transition to Open Access may be longer than 1 January 2020, but as short as possible and respecting the targets already set by the individual research funders.

## cOAlition S: building an alliance of funders and stakeholders

Plan S states the fundamental principles for future Open Access publishing. Science Europe, funders, the European Research Council and the European Commission will work together to clarify and publish implementation details. The plan does not advocate any particular Open Access business model, although it is clear that some of the current models are not compliant. We therefore invite publishers to switch to publication models that comply with these principles.

Plan S was initiated by the Open Access Envoy of the European Commission and further developed by the President of Science Europe and by a group of Heads of national funding organisations. It also drew on substantial input from the Scientific Council of the European Research Council.

Today, a group of national funders initiate the alliance cOAlition S (http://scieur.org/coalition-s) to take action towards the implementation of Plan S, and are joined by the European Commission and the European Research Council.

We invite other funding agencies and research councils, as well as stakeholders (notably researchers, universities, libraries, and publishers) to join cOAlition S and thereby contribute to the swift realisation of our vision of science without publication paywalls.
